# 

**Published:** 1857-09

**Authors:** 


NORTH AMERICAN

### MEDICO-CHIRURGICAL REVIEW.

NO. 5.—SEPTEMBER, 1857.

### CONTENTS.

### ANALYTICAL AND CRITICAL REVIEWS.

PAGE

ART. I.—1. Réflexions sur l'Intermittence considérée chez l'homme dans l'état de Santé et dans l'état de Maladie, &c. Par E. PALLAS, M.D., . . . . . . . . . . . . 641

2. De la Périodicité des Fièvres Intermittentes et des Causes qui la Produisent. Par M. F. M. AUDOUARD, D.M.M., . . . 641

3. Des Irritations Nerveuses sous le Rapport de la Thérapeutique. Par GUERIN DE MAMERS, D.M.P., . . . . . 641

4. Des Fièvres Intermittentes sous le Rapport de l'Etiologie et de la Thérapeutique. Par GUERIN DE MAMERS, D.M.P., . . 641

5. Traité Anatomico-Pathologique des Fièvres Intermittentes Simples et Pernicieuses. Par E. M. BAILLY, D.M.P., . . 641

ART. II.—1. Indigenous Races of the Earth, or New Chapters of Ethnological Inquiry. By J. C. NOTT, M.D., and GEO. R. GLIDDON, formerly U. S. Consul at Cairo, . . . . . . . 665

2. Crania Britannica. By JOSEPH BARNARD DAVIS, M.R.C.S., F.S.A., &c., and JOHN THURNAM, M.D., F.S.A., . . . . 665

3. Essai sur les Déformations Artificielles du Crane. Par L. A. GOSSE, de Genève, Docteur en Médecine, &c., . . . 665

4. Schædel, Hirn und Seele des Menschen und der Thiere nach Alter, Geschlecht und Race, dargestellt nach neuen Methoden und Untersuchungen von EMIL HUSCHKE, . . . . . 665

ART. III.—A Treatise on Cancer, and its Treatment. By J. WELDON FELL, M.D., . . . . . . . . . . . . . 688

ART. IV.—An Exposition of the Signs and Symptoms of Pregnancy. By W. F. MONTGOMERY, A.M., M.D., M.R.I.A., . . . . . 694

ART. V.—On the Diseases of Women; including those of Pregnancy and Childbed. By FLEETWOOD CHURCHILL, M.D., T.C.D., M.R.I.A., 695

ART. VI.—Smithsonian Contributions to Knowledge. By JOSEPH JONES, M.D., Professor of Chemistry in the Savannah Medical College, 697

ART. VII.—Leçons de Physiologie Expérimentale, Appliquée la Médecine, faites au Collége de France, &c. Par M. CLAUDE BERNARD, Membre de l'Institut de France. Prof. de Médecine au Collége de France. Prof. de Physiologie à la Faculté des Sciences, &c. &c., . . . . . . . . . . . . . 699

PAGE

ART. VIII.—Elements of Pathological Anatomy. By SAMUEL GROSS, M.D., Professor of Surgery in the Jefferson Medical College of Philadelphia, &c., . . . . . . . . . . . 702
ART. IX.—Manual of Physiology. By WILLIAM SENHOUSE KIRKES, M.D., F.R.C.P., &c., . . . . . . . . . . . 704

### ORIGINAL COMMUNICATIONS.

ART. I.—Remarks upon the State of Medical Science, and Some of the Diseases of Syria and Arabia. By R. GUTZLAFF BARCLAY, M.D., . . . . . . . . . . . . . . . 705
ART. II.—The Parent Tissue and the Blastema. By W. S. BOWEN, M.D., of New York, . . . . . . . . . . 719
ART. III.—On the Excretion of Phosphoric Acid by the Kidneys. By WILLIAM A. HAMMOND. M.D., U.S.A., . . . . . . 730
ART. IV.—Case of Ovarian Tumor; Excision; Death; By M. KEMPF, M.D., of Ferdinand, Indiana, . . . . . . . . . . 738
ART. V.—A Case of Pregnancy in a Female, in whom the Menstrual Function had been for some years suspended. By O. C. GIBBS, M.D., of Frewsburg, New York, . . . . . . . 741
ART. VI.—Case of Lamellar Cataract, with Remarks. By E. WILLIAMS, M.D., of Cincinnati, . . . . . . . . . . . 743
ART. VII.—Pathology and Treatment of Anæmia. By H. P. AYRES, M.D., of Fort Wayne, Indiana, . . . . . . . 748

### BI-MONTHLY PERISCOPE.

### SURGERY.

1. Treatment of Aneurism by Manipulation, . . . . . . . 755
2. Professor Bœck on Syphilization, . . . . . . . . . . 758
3. Case of Excision of the Os Calcis, by an Improved Method of Operating, 762
4. Selections from the unpublished Manuscripts of the late Abraham Colles, 763
5. On the Concretions of the Prostate, . . . . . . . . . 769

### PRACTICAL MEDICINE.

1. Notes on the Use of Glycerine in Consumption, . . . . . . 770
2. Lactic Acid a Remedy for Dyspepsia, . . . . . . . . . 772

### THERAPEUTICS AND MATERIA MEDICA.

1. Report of a Case of Death caused by swallowing Oil of Turpentine, . 772
2. On the Employment of Amylene, . . . . . . . . . 775

### OBSTETRICS.

1. Secondary Uterine Hemorrhage, . . . . . . . . . . 778
2. On the Use of the Speculum, . . . . . . . . . . . 783
3. Practical Observations on Blistering the Cervix Uteri, as a Remedial Agent in the Treatment of certain Diseased Conditions of that Viscus, 783
EDITORS' TABLE, . . . . . . . . . . . . . . . 787
BI-MONTHLY BIBLIOGRAPHICAL RECORD, . . . . . . . . . 800

